# Sensorimotor Cortex Reorganization in Alzheimer's Disease and Metal Dysfunction: A MEG Study

**DOI:** 10.1155/2013/638312

**Published:** 2013-12-12

**Authors:** C. Salustri, F. Tecchio, F. Zappasodi, L. Tomasevic, M. Ercolani, F. Moffa, E. Cassetta, P. M. Rossini, R. Squitti

**Affiliations:** ^1^Institute of Cognitive Sciences and Technologies (CNR), Unità MEG, Fatebenefratelli Hospital, Isola Tiberina, 00186 Rome, Italy; ^2^Department of Imaging, IRCCS San Raffaele Pisana, 00163 Rome, Italy; ^3^Department of Neuroscience and Imaging, Gabriele d'Annunzio University, 66100 Chieti, Italy; ^4^AFaR, Department of Neuroscience, Fatebenefratelli Hospital, Isola Tiberina, 00186 Rome, Italy; ^5^Institute of Neurology, Department of Neuroscience, Catholic University, A. Gemelli Polyclinic, 00168 Rome, Italy; ^6^Laboratory of Neurodegeneration, IRCCS San Raffaele Pisana, 00163 Rome, Italy

## Abstract

*Objective*. To verify whether systemic biometals dysfunctions affect neurotransmission in living Alzheimer's disease (AD) patients. *Methods*. We performed a case-control study using magnetoencephalography to detect sensorimotor fields of AD patients, at rest and during median nerve stimulation. We analyzed position and amount of neurons synchronously activated by the stimulation in both hemispheres to investigate the capability of the primary somatosensory cortex to reorganize its circuitry disrupted by the disease. We also assessed systemic levels of copper, ceruloplasmin, non-Cp copper (i.e., copper not bound to ceruloplasmin), peroxides, transferrin, and total antioxidant capacity. *Results*. Patients' sensorimotor generators appeared spatially shifted, despite no change of latency and strength, while spontaneous activity sources appeared unchanged. Neuronal reorganization was greater in moderately ill patients, while delta activity increased in severe patients. Non-Cp copper was the only biological variable appearing to be associated with patient sensorimotor transmission. *Conclusions*. Our data strengthen the notion that non-Cp copper, not copper in general, affects neuronal activity in AD. *Significance*. High plasticity in the disease early stages in regions controlling more commonly used body parts strengthens the notion that physical and cognitive activities are protective factors against progression of dementia.

## 1. Introduction

In the last decade, increasing evidence has revealed the role of biometals dysfunctions in Alzheimer's disease (AD) (see [1] for a review). AD is characterized by a progressive degradation of cognitive abilities, inexorably leading to dementia, due to a gradual loss of neurons and synapses. Also sensori-motor deficits are characteristic of the disease but they usually appear in the late severe stages of the disease.

Topographic and frequency changes of the sensorimotor cortex's electro- and magneto-encephalographic activities have been observed to follow the progression of the disease [2, 3]. These changes are not attributable to lesion densities, since the density of plaques and tangles in the sensorimotor cortex of AD brains has been shown to be equivalent to that of other brain areas more specific to AD [4]. Evidently, other phenomena play a role and one of them is likely to be the sensorimotor cortex's formidable capability to reorganize its circuitry damaged by AD degeneration into new and still healthy locations [3, 5, 6].

The aim of the present paper was to investigate reorganization within the primary sensorimotor cortex in AD and verify the role of systemic metal derangements in this reorganization. We studied both rest and stimulus-evoked magnetoencephalographic (MEG) activities in the sensorimotor cortex of a group of AD patients and a matched group of controls. The study of the spontaneous activity focused on the oscillatory rhythms, while we investigated the evoked activity focusing on the first two components of the magnetic somatosensory Evoked Fields (SEFs), M20 and M30, recorded during stimulation of the median nerve. M20 is well known to represent the first synchronous activation of Brodmann area 3b, when the contralateral median nerve is stimulated [7]. It is stable, highly repeatable, and completely independent of subject's attention [8]. It is mediated by glutamate neurotransmission [9, 10]. M30, instead, appears to be generated by a joint contribution of the motor and somatosensory cortices and is mediated by a major contribution of both glutamate and Gaba neurotransmitters. Levels of copper, ceruloplasmin, non-Cp copper, peroxides, transferrin, and total antioxidant (TAS) capacity were correlated with M20 and M30 indices in both healthy and AD subjects.

## 2. Materials and Methods 

The study was approved by the local ethics committee and each participant or caregiver signed an informed consent.

### 2.1. Subjects

Sixteen patients, age 55 to 86 years (mean 73 ± 7 years, 13 females), were enrolled in the study. “Probable AD" had been diagnosed in all patients according to NINCDS-ADRDA criteria [11]. Patients underwent general medical, neurological, and psychiatric assessments and were rated with standard neuropsychological instruments which included the mini mental state examination (MMSE) [12], the clinical dementia rating scale [13], the geriatric depression scale [14], the Hachinski ischemic scale [15], and the instrumental activities of daily living [16]. They also underwent neuroimaging diagnostics and standard laboratory analyses to rule out other causes of dementias. Exclusion criteria were (i) fronto-temporal dementia; (ii) vascular dementia; (iii) extra-pyramidal syndromes; (iv) Parkinson's disease; (v) reversible causes of dementias; (vi) Lewy body dementia. Detection of vascular burden was based on guidelines previously developed within our clinical network [17].

The control sample was made of 16 subjects (7 females) age-matched to the AD patients (mean age 72 ± 14 years, two-tailed *t*-tests *P* = 0.801). All controls underwent physical, neurological, and neuropsychological examinations.

### 2.2. MRI Investigation

Brain MRI was performed at 1.5 tesla. The imaging protocol consisted of axial T1W spin echo and T2W double Spin Echo sequences in axial, coronal, and sagittal planes, with 5 mm slice thickness and interslice gap = 0.5 mm. MR images were evaluated by two experienced neuroradiologists blind to the patients' diagnoses or laboratory results, who approached total agreement (95%). Atrophy was graded following standard visual rating scales on plain MRI. The degree of medial temporal lobe atrophy was evaluated with a ranking procedure and validated by linear measurements of the medial temporal lobe including the hippocampal formation and surrounding spaces occupied by CSF, following the standard five-point rating scale of medial temporal lobe atrophy (MTA). Generalized brain atrophy (ventricular and sulcal atrophy) was rated as absent (=0) and present (=1). It must be noted that some degree of atrophy is normally present in the old age. Thus, atrophy was classified as absent when falling within the moderate levels shared by all elderly individuals.

### 2.3. MEG Investigation

A 28-channel MEG system [18], covering a total scalp area of about 180 cm^2^, operating inside a magnetically shielded room (Vacuumschmelze GMBH), was used to record brain magnetic fields from left and right hemispheres. The sensors were positioned over the area of the contralateral sensorimotor cortex controlling the hand (C3/C4 site of the International 10–20 EEG system). Subjects lay on a nonmagnetic bed, with their eyes open to reduce disturbance by alpha activity. The recording procedure lasted about 30 min for all subjects.

Magnetic evoked fields were recorded under electrical stimulation of the contralateral median nerve at the wrist by 0.2 ms electric pulses (cathode proximal), with 631 ms inter-stimulus interval. Stimulus intensities were individually set just above the threshold inducing a painless twitch of the thumb.

Spontaneous brain activity with eyes open was recorded continuously for three minutes on each hemisphere.

Signals were bandpass filtered between 0.48–250 Hz, sampled at 1 kHz and stored for off-line process.

### 2.4. Biochemical Investigations

Sera from overnight fasting blood samples were drawn in the morning and rapidly stored at −80°C. Biochemical variables were determined according to the established methods reported in details elsewhere [19]. Briefly, serum copper concentration was measured following the method of Abe et al. [20] (Randox Laboratories, Crumlin, UK) and with an Analyst 600 Perkin Elmer atomic absorption spectrophotometer equipped with a graphite furnace with platform HGA 800. Transferrin [21] and ceruloplasmin [22] were measured by immunoturbidimetric assays (Horiba ABX, Montpellier, France). The iron level in serum was determined using Ferene colorimetric method (Horiba ABX, Montpellier, France). All biochemical measures were automated on a Cobas Mira Plus analyzer (Horiba ABX, Montpellier, France) and performed in duplicate. For each serum copper (total copper) and ceruloplasmin pair we computed the amount of copper bound to ceruloplasmin (CuB) and the amount of copper not bound to ceruloplasmin (free copper) following standard procedures [23]; briefly: CuB = ceruloplasmin (mg/dL) ∗ 10 ∗ *n*; *n* = 0.0472 (*μ*mol/mg); free copper = total copper-CuB). This calculation expresses free copper in *μ*mol/L and is based on the fact that ceruloplasmin contains 0.3% of copper [23]. Thus, for a subject with a serum copper concentration of 17.3 *μ*mol/L and a serum ceruloplasmin concentration of 33 mg/dL, the bound copper concentration was 33 ∗ 10 ∗ 0.0472 = 15.6 *μ*mol/L, and the free copper concentration 17.3 − 15.6 = 1.7 *μ*mol/L.

### 2.5. Data Analysis

An *ad hoc* developed semiautomatic (i.e., partially based on visual inspection) artifact-rejection procedure [24] was first applied to all data to detect and discard the contribution of spurious sources, such as heart, eyes, and muscles, which normally generate signals in the same frequency range of the sources under study. About 280 artifact-free trials were filtered between 3–150 Hz and averaged to obtain the time course of the magnetic evoked fields. The signal amplitude was measured as the deviation from a baseline defined as the mean value of the signal in the 5–15 ms poststimulus window.

**Figure 1:**
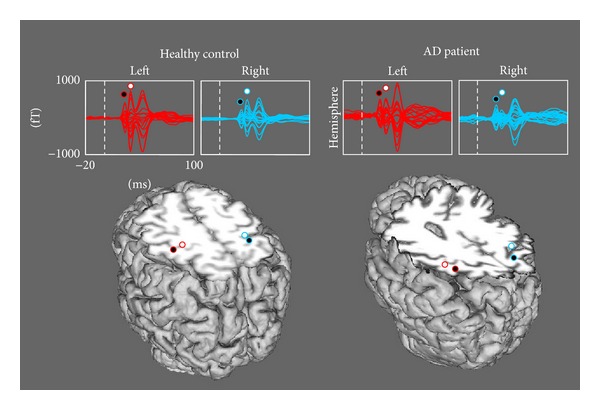
SEF and M20 and M30 ECD positions in a representative healthy control (*F*, 68 y.o., MMSE = 29) and an AD patient (*F*, 63 y.o., MMSE = 24). (Top) superimposition of all channels in the rolandic region, averaged in the [−20, 100] ms period, 0 representing the time of stimulus delivery. The amplitude of the magnetic field is represented in femtotesla (fT) on the *y*-axis; (Bottom) positions of the ECDs explaining the cerebral activation in correspondence to the M20 (black circle) and M30 (white circle) components, projected onto a suitable axial slice after volumetric head reconstruction from individual MR images.

**Figure 2:**
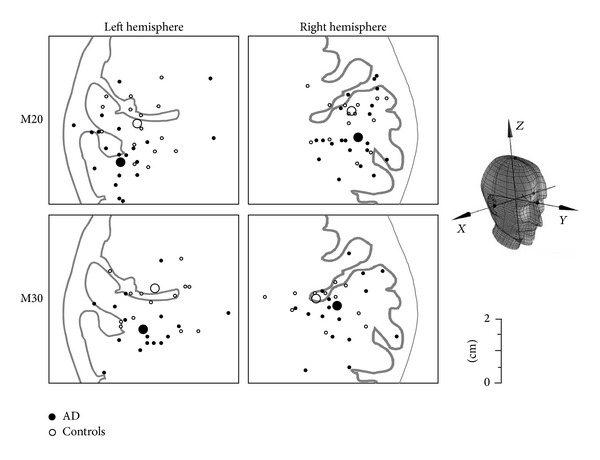
M20 and M30 ECD positions. Positions of individual M20 (top) and M30 (bottom) ECDs in healthy controls (empty dot) and AD patients (black dot) and of the average across the two groups (larger circles) projected on a representative axial graphic rendering in the region of the hand control. *Right side*: spatial coordinate system: the *y*-axis runs through the midpoint between the subject's preauricular points and his/her nasion; the *z*-axis is the line perpendicular to the *y*-axis that passes through the vertex; the *x*-axis is the line perpendicular to both *y* and *z*-axes that runs through their intersection. The intersection of the *x*-, *y*- and *z*-axes is the origin of the subject-based coordinate system, and *x* is positive on the head's right side, *y* towards the nasions and *z* towards the vertex.

M20 and M30 were identified by the maxima of the global field power within time windows centered respectively at 20 and 30 ms after stimulus delivery (Figure 1(a)). Latency, spatial coordinates and strength of the equivalent current dipoles (ECDs) generating M20 and M30 magnetic fields were computed by solving the inverse problem in the model of a dipole moving inside a homogeneously conducting sphere. ECD localizations were accepted only if they explained at least 90% of the variance. In our coordinate system (Figure 2), we defined as *displacement* the Euclidean distance between the patients' ECDs positions and the center of mass of the controls' ECDs.

Interhemispheric asymmetries were assessed by measuring the Euclidean distance between each component's left and right ECD positions, after relocating them in the same hemisphere (i.e., measuring in the left hemisphere after mirroring the right ECD position into the left hemisphere or vice versa).

Spectral characteristics of the background spontaneous activity were evaluated through the signal power spectral density (PSD), estimated for each MEG channel following the Welch procedure (2048 ms duration, Hanning window, 60% overlap, and about 180 artifact free trials used). The PSD mean of the 16 inner axial gradiometers, covering a circular area of about 12 cm diameter, was taken as the total PSD. Total signal power was computed by integrating the total PSD in the 2–44 Hz frequency interval. Spectral properties were investigated in the standard frequency bands: 2–3.5 Hz (delta), 4–7.5 Hz (theta), 8–12.5 Hz (alpha), 13–33 Hz (beta) and 33.5–44 Hz (gamma).

An MEG-MRI common reference system was used based on three head landmarks corresponding to nasion, left, and right preauricular points [25] (Figure 2). The MRI procedure lasted about 30 min and was well tolerated by all patients.

### 2.6. Statistical Analysis

M20 and M30 ECD coordinates and displacements displayed a Gaussian distribution (Kolmogorov-Smirnov test, *P* > 0.2). Absolute power values showed a Gaussian distribution after log transformation (*P* > 0.2).

In order to compare characteristics between patients and controls, ANOVA for repeated measures was applied to ECD latencies, positions and displacements, including *Hemisphere* (left, right) as within-subject factor and *Group* (patient, control) as between-subject factor. The 3-dimensional dipole coordinate vectors (*x*, *y*, and *z*) were used for the positions. The factor *Band* (delta, theta, alpha, beta, and gamma) was used when we analyzed the spontaneous activity spectral characteristics in left and right rolandic regions.

In order to pinpoint differences due to global atrophy patients were divided in two subgroups (atrophy, no-atrophy). ANOVA was then applied separately to ECD positions, displacements and delta and theta powers, with *Hemisphere* (left, right) as within-subjects and *Global Atrophy* (atrophy, no-atrophy) as between-subjects factors.

To pinpoint differences due to levels of illness progression, patients were divided in two subgroups, one including moderately ill patients (MMSE 25–20) and the other including severely ill patients (MMSE < 20). ANOVA was then applied separately to ECD displacements and delta and theta powers, with *Hemisphere* (left, right) as within-subjects and *Illness Severity* (severe, moderate, and controls) as between-subjects factors.

Inter-hemispheric asymmetries of the ECD positions were studied by ANOVA with *Component* as within-subjects and *Group* (patients, controls) as between-subjects factors.

## 3. Results

### 3.1. MEG Analysis of Plastic Phenomena

#### 3.1.1. M20 and M30 ECDs Recruited by Median Nerve Stimulation

Table 1 shows the means of M20's and M30's latencies, position coordinates, and ECD strengths in patients and controls.

**Figure 3:**
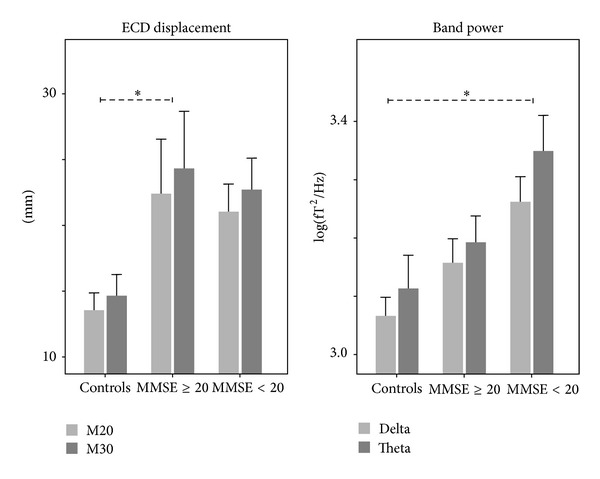
Patients' clinical severity in relationship with left displacements and rest low band powers. Mean and standard errors in controls and patients are subdivided in moderate (MMSE 25–21) and severe (MMSE ≤ 20) clinical severity. *Left*: M20 and M30 ECD in the left hemisphere. *Right*: delta and theta bend powers of left and right activity in resting state.

The M20 and M30 ECD positions of our patients resulted shifted with respect to the ones of our controls, as indicated by the ANOVA *Group* effect applied to the dipoles' coordinate vectors (*F*(3,18) = 4.146, *P* = 0.021) (Figures 2 and 3 left). In particular, the patients' M20 and M30 appeared more posterior in both hemispheres, although the shift reached statistical significance only in the left hemisphere (post-hoc *y*-coordinate *t*-test: *P* = 0.04 and 0.02 for M20 and M30, resp.).

The sources' displacements showed evident differences between patients and controls in both hemispheres as indicated by the *Group* effect (*F*(1,20) = 6.587, *P* = 0.018). Again, the difference resulted statistically significant only in the left hemisphere (Table 2). Inter-hemispheric asymmetries of ECD locations did not differ in patients and controls (*Group* effect *P* > 0.5, with no interaction with *Component*).

ANOVA applied to ECD's strengths and latencies showed no *Group* effect.

### 3.2. Spontaneous Activity

Since ANOVA applied to Rolandic rest activity showed a strong *Band* ∗ *Group* effect (*F*(2.4,59.6) = 7.052; *P* = 0.001), we repeated the analysis separately for each frequency band. A *Group* effect was found in the delta (*F*(2,24) = 5.577; *P* = 0.010) and theta (*F*(2,24) = 3.383; *P* = 0.051) bands. Patients showed a higher power in low-frequency bands than controls in both hemispheres.

To test whether the spatial displacements observed in AD patients were due to structural displacements secondary to cortical atrophy, we investigated whether any rest activity was generated from cortical regions more frontal than the M20 generator. We considered the cortical patches 1.5 cm anterior and posterior to the central sulcus, and estimated the percentage of background activity dipoles falling in this spatial range both in patients and controls. A general linear model with *Hemisphere* (left, right) as within-subjects factor and *Group* (patients, controls) as between-subjects factor revealed no significant difference (*P* > 0.2).

### 3.3. Relationship of Cortical Displacements and Background Activity with Brain Atrophy

No dependence on *Global Atrophy* was shown by the patients' M20 and M30 ECD positions (*F*(3,13) = 1.482, *P* = 0.266), sources' displacements (*F*(1,15) = 0.058, *P* = 0.813), and delta and theta band powers (*F*(1,12) = 0.062, *P* = 0.807).

Middle temporal atrophy showed no nonparametric correlation with either any cortical displacements or background activity variable or clinical severity (*P* > 0.200 consistently).

Furthermore, clinical severity did not depend on *Global Atrophy* (independent *t*-test *t*(15) = 0.828, *P* = 0.421).

### 3.4. Relationship of Cortical Displacements and Background Activity with Clinical Status

ANOVA revealed a significant *Illness Severity* effect for displacements in the left hemisphere (*F*(2,20) = 5.005, *P* = 0.017, Figure 3 right). Post-hoc comparison (Bonferroni corrected) revealed a significant difference between controls and moderate patients (*P* = 0.032). ANOVA on spectral background activity showed a significant *Illness Severity* effect for low band powers (delta: *F*(2,24) = 5.577, *P* = 0.010; theta: *F*(2,24) = 3.383, *P* = 0.051). Bonferroni post-hoc comparison revealed a difference between controls and severe patients (delta: *P* = 0.016, theta: *P* = 0.047, Figure 3 right). Displacements differences between controls and severe patients (*P* > 0.200) were not significant. Also delta and theta powers differences between controls and moderate patients (*P* > 0.200) were not significant.

#### 3.4.1. Cortical Excitability and Metal and Oxidative Stress Derangement

The biological variables under study did not correlate with age and sex.  Table 3 shows the correlation among M20 and M30 ECD strengths as markers of glutamate S1 and glutamate-Gaba SM1 excitability in healthy controls and patients affected by AD. While in healthy controls M20 (s20II) was mainly associated with ceruloplasmin and peroxides [26], in AD these relationships were not evident. Instead, a significant association appeared between higher M30 ECD strength (s30II) and non-Cp copper, which is absent in healthy controls.

## 4. Discussion

The main result of this study is that non-Cp copper is associated with the primary sensorimotor cortical excitability in AD patients and S1 area of the dominant hemisphere displays plastic changes in the early phase of the disease.

As we did previously with healthy subjects [26], we focused our study on a brain circuit which connects pyramidal neurons in the somatosensory cortex with a projection coming from neurons in the thalamus. We showed that a marker of glutamate-mediated cortical excitability, that is, the M20 ECD strength, is associated with the concentrations of a marker of copper status, that is, ceruloplasmin, in healthy controls, while the M30 ECD strength is associated with higher concentrations of non-Cp copper in AD patients.

We focused a previous study on systemic metal variations in healthy subjects in relation with indices of glutamatergic neurotransmission, observing that the higher the ceruloplasmin the lesser the cortical glutamatergic neurotransmission as revealed by MEG. Conversely, in AD patients, our results demonstrate that ceruloplasmin is not associated with either M20 or M30 ECD strength, differently from non-Cp copper. The shift in the correlation of this metal indices of copper status appears related to the diverse nature of the two copper components analysed. While ceruloplasmin is a protective factor in many diverse conditions, non-Cp copper still remains a toxic component of copper metabolism, specifically related to the AD condition. The fact that the AD brain is under the threat of oxidative stress, also mediated by metals via Haber Weiss and Fenton's chemistry, can be one of the mechanisms subtending the plastic phenomena presently detected. More specifically, we observed a spatial shift of the AD patients' sensorimotor generators with no change of latencies or strength and with neuronal reorganization more evident in moderate AD patients. Conversely, in severe patients delta activity increased, as previously observed [2, 3]. Plastic phenomena were more evident in the patients' dominant hemisphere, which is more used for daily activities. These plastic phenomena suggest that the cortex delays the appearance of evident deficits in the attempt of escaping the advancing neuronal degeneration, either by utilizing previously silent networks or by establishing brand new circuitries. Conceivably, symptoms appear when reorganization is overwhelmed by neurodegeneration. Non-Cp copper derangement, as we observed in AD, is among the biological processes underlying cortex activity reorganization.

We showed that the cortical sources of the earliest responses to contralateral median nerve stimulation are shifted posteriorly in AD patients with respect to controls and that these shifts are not due to brain atrophy. When the median nerve is stimulated at the wrist, the axonal signal traveling towards the brain reaches the ventroposterior lateral thalamic nucleus (VPL), which relays the majority of the information to areas 3a and 3b of the sensory cortex. Area 3a is invisible to MEG, due to the orientation of its pyramidal cells perpendicular to the scalp, but it quickly transfers information to the neighboring area 3b, whose pyramidal cells are instead parallel to the head surface [27, 28]. This cortico-cortical contribution adds to the activation generated by the signal arriving to area 3b directly from the VPL and the two produce a strong and reliable MEG signal, represented by M20. M30 is more puzzling. Some authors localized it in area 3b [29], while others in motor area 4 [30].

Our results showed no differences between AD patients and controls in M20's and M30's latencies and amplitudes (*P* > 0.2), an evidence that stresses the role of plasticity: since thalamic pathways to the cortex are relatively spared in AD [31], the amount of information relayed to the cortex remains approximately unaltered while neurodegeneration ravages the corticocortical interconnections, on which both M20 and M30 partially rely. This result appears in contrast with a recent work by Stephen et al. [32], who instead observed a significant diagnosis-dependent difference of the amplitude. However, they observed this difference only in their male participants, although the wider part of their patient population consisted of females. The difference between the two studies possibly originates from the fact that we did not differentiate on the basis of sex and that we did not consider successive recruitments. We studied the single response separately, since we were interested in assessing the behavior of a specific portion of cortical activity.

The parietal direction of M20's drift was probably to be expected from the fact that the sulcus lies immediately ahead and that the VPL projections are mainly directed towards parietal regions. Nonetheless, our displacement results (Table 2), which show a much wider localization spread in patients than controls, indicate that occasional recruitments within the motor cortex cannot be ruled out. In the case of M30, we speculate that the shift of its ECDs could be interpreted as a sign of a gradual loss of the motor contribution to the component.

The fact that plasticity appeared stronger in our patients' dominant hemisphere suggests that it works to enhance preservation of the more common right-hand activities. This notion is in line with epidemiological data showing that individuals engaging in higher levels of mental and physical activity are at lower risk of developing AD and dementias (review in [33]). Regular activity increases the endurance of cells and tissues to oxidative stress and influences vascularisation, energy metabolism, and neurotrophin synthesis, which all play an important role in neurogenesis and plasticity.

The nature of our investigation does not allow definite statements on the actual mechanisms subtending the described plastic changes. The sensorimotor cortex may activate a number of mechanisms including changes in neuronal membrane excitability [34], strengthening [35], or weakening [36] of existing synapses, creation of new synapses [37], and even “unmasking” areas normally kept silent by tonic inhibition [38]. All these mechanisms are certainly not mutually exclusive. Since glutamate is the major excitatory neurotransmitter involved in sensory and motor thalamo-cortical projections, we can conceive that the glutamatergic system is mainly involved in the plasticity phenomena that we observed. This system suffers major disruptions in AD especially due to synaptic malfunctions and apoptosis, together with neurotransmitter demodulation. The correlation of non-Cp copper with M30 and not with M20 ECD strength suggests that complex neurotransmission is involved in the non-Cp copper dysfunction.

Finally, while plasticity phenomena appeared more evident in less impaired AD patients, their spontaneous activity showed a linear behavior with disease severity (Figure 3). The power of both delta and theta rhythms increased with increasing illness severity. A bulk of evidence from different approaches shows that the spectral characteristics of the electromagnetic background brain rhythms significantly reflect network functioning within various brain regions, especially in AD [39]. Discrimination between physiological and pathological brain aging clearly emerges at the group level, with low frequencies within the delta/theta range marking the neural degeneration. Delta dipole distributions used to estimate the neuronal sources in diverse regions of AD brains have shown increased densities in posterior temporal areas [40]. This topographic change in brain activity possibly reflects changes in the cholinergic system of AD brains, a view supported by the evidence that administration of scopolamine, an antagonist of muscarinic receptors, to healthy elderly subjects induces alterations of their spontaneous oscillatory activity similar to the ones seen in AD [41]. Functional deficits due to cholinergic dysfunctions are typical of the late stage of AD.

In summary, this study showed that the copper dysfunction typical of AD patients is associated with cortical excitability, both glutamate- and gaba-mediated, within the peripheral-central neural sensorimotor pathways.

## Figures and Tables

**Table 1:** M20 and M30 ECD characteristics in patients and controls.

	Left hemisphere					Right hemisphere				
	*t* (ms)	*x* (mm)	*y* (mm)	*z* (mm)	*s* (nA m)	*t* (ms)	*x* (mm)	*y* (mm)	*z* (mm)	*s* (nA m)
M20										
Patients	21.3 ± 2.0	−48,9 ± 8,7	−20,9 ± 10.7	64,3 ± 15,6	17.9 ± 7.8	21.3 ± 1.9	48,5 ± 9,0	−14,5 ± 10,2	70,3 ± 13,9	15.7 ± 6.5
Controls	22.3 ± 1.7	−44,2 ± 7,0	−13,4 ± 9,3	67,7 ± 9,3	21.8 ± 11.6	22,3 ± 2.3	44,9 ± 7,5	−11,4 ± 9,8	71,7 ± 9,0	19.2 ± 8.0
M30										
Patients	28.9 ± 3.4	−37,6 ± 25,4	−21,5 ± 8,8	65,7 ± 15,3	29.2 ± 17.8	29.6 ± 4.6	41,9 ± 8,0	−19,7 ± 11,9	69,5 ± 15,1	26.3 ± 14.4
Controls	30.5 ± 3.4	−39,8 ± 9,0	−13,5 ± 7,3	69,1 ± 9,0	29.1 ± 18.4	30.3 ± 4.0	38,6 ± 9,4	−14,6 ± 7,9	74.8 ± 8,3	26.4 ± 16.4

Mean and standard deviation of latencies (*t*), positions (*x*, *y*, *z*) and strength (*s*) of equivalent current dipoles explaining M20 and M30 magnetic field distributions (see methods).

**Table 2:** M20 and M30 ECD displacements.

	M20		M30	
	Left *H*	Right *H*	Left *H*	Right *H*
Controls	13.3 ± 5.9	13.5 ± 6.4	13.1 ± 5.5	12.9 ± 6.3
Patients	21.3 ± 7.0	17.7 ± 8.5	19.1 ± 8.1	23.2 ± 21.2
*P*	**0.001**	0.132	**0.037**	0.121

Mean ± standard deviation of the displacements (mm) of M20 and M30 sources in the two hemispheres (*H*) for controls and patients. The last raw shows the *P* value of two-tailed independent *t*-test between the two groups.

**Table 3:** Correlation between SM1 excitability and metal variables.

	Healthy control		AD patients	
	M20 ECD strength	M30 ECD strength	M20 ECD strength	M30 ECD strength
Copper	−**.760**	.486	−.010	.071
	***.011***	*.222 *	*.973 *	*.826 *
Perox	−**.823**	.058	−.351	−.338
	***.003***	*.891 *	*.239 *	*.282 *
Cerulopasmin	−**.882**	.136	−.463	−.539
	***.002***	*.771 *	*.111 *	*.070 *
Transferrin	−.514	.017	−.461	−.559
	*.193 *	*.975 *	*.180 *	*.118 *
TAS	−.054	.290	−.043	.059
	*.890 *	*.528 *	*.888 *	*.855 *
Non-Cp copper	−.357	.561	.393	**.648**
	*.345 *	*.190 *	*.184 *	***.023***

Pearson's coefficient (upper row) and two-tail significance (lower row) of the correlation between the metal variables and the ECD strengths (expressed as nA∗m) of glutamate-mediated M20 and glutamate- and gaba-mediated M30 for controls and patients. Bold font is used when *P* < 0.05.
